# Management of a patient with Huntington’s disease under spinal anaesthesia

**DOI:** 10.4103/0019-5049.65352

**Published:** 2010

**Authors:** Sanjeev Janardan Juwarkar, Chitra Sanjeev Juwarkar

**Affiliations:** Vintage Hospital and Medical Research Center, Panaji; 1 Department of Anaesthesia, Goa Medical College, Goa

Sir,

Huntington’s disease (HD), with a prevalence of 10 cases per million worldwide is a fatal autosomal dominant disorder characterised by progressive motor, emotional and cognitive dysfunction caused by mutation in the Huntington’s gene. The onset is typically between 35 and 45 years and the duration averages 17 years from the time of diagnosis to death.[1]

The clinical diagnosis can be readily made in cases with positive family history of an insidious onset of chorea, dementia[2] and emotional symptoms.

We share our experience of managing such a rare case in our day-to-day practice.

A 44-year-old, 70 kg, female, mother of two, with dysfunctional uterine bleeding was posted for endometrial ablation. The patient was apparently fine prior to 10 years, when she noticed increased blinking of eyes and some facial movements, which gradually spread and involved her whole body since the last three to four years. The movements were more in the extremities and patient could not sit still in one place. The movements were less during sleep. She would do most of her household work. This patient with normal vitals and a normal cardiorespiratory system had apparently normal higher functions.

As the procedure was restricted to the uterus we decided to conduct neuraxial blockade. The patient was prepared for the procedure after explaining the details. Oral diazepam 5 mg and IV ondensetron were administered 45 minutes prior. Continuous arm movements made securing of the IV line a difficult task; it was then doubly fixed and fluids infused. The monitors were firmly attached. Her continuous body movements made it difficult for us to give her the required position for spinal anaesthesia. She was made to sit at the edge of the operating table with her legs hanging down and two OT attendants holding her firmly to make lumbar puncture possible. We could get dural puncture at the very first instant, with a 25 gauge needle. 2.8 ml of heavy bupivacaine was then injected to achieve analgesia up to the T8 level. The procedure lasted 50 minutes. Oxygen was administered and no sedative or hypnotic was given. Vitals were stable throughout and no vasopressors were used. She continued to have movements of both her arms and head throughout the procedure, but not of the lower extremities. In the recovery room, she gradually regained the sensations in the anaesthetised parts and within three-and-a-half hours the preoperative movements appeared in the lower limbs. She had an uneventful hospital stay till her discharge on the fourth postoperative day.

There are few case reports published describing the anaesthetic management of patients with Huntington’s chorea.[3,4] Major concerns in management are potential difficult airway, sleep apnoea, risk of aspiration[1] and altered reactions to various drugs. For general anaesthesia, propofol, short-acting neuromuscular agents, nitrous oxide, sevoflurane combined with an opiod and droperidol are safe. Although reported experience with regional analgesia is sparse, spinal anaesthesia has been successfully administered.[5]

Reviewed literature showed a higher possibility of complications with general anaesthesia; hence we opted for a neuraxial blockade. The movements of lower limbs slowly reappeared once the effect of the drug wore off. This could be explained, as the pathology in HD lies in the brain and all the pathways are blocked when the neuraxial block is achieved.
